# Rapidly Growing Thyroid Mass: An Unusual Case of Acute Lymphoblastic Leukemia

**DOI:** 10.4274/tjh.2015.0136

**Published:** 2015-08-01

**Authors:** Füsun Özdemirkıran, Gonca Oruk, Bahriye Payzin, Zeynep Gümüş, Melike Koruyucu, Türkan Atasever, Betül Küçükzeybek, Özgür Esen

**Affiliations:** 1 Katip Çelebi University Faculty of Medicine, Atatürk Training and Research Hospital, Clinic of Hematology, İzmir, Turkey; 2 Katip Çelebi University Faculty of Medicine, Atatürk Training and Research Hospital, Clinic of Endocrinology, İzmir, Turkey; 3 Katip Çelebi University Faculty of Medicine, Atatürk Training and Research Hospital, Clinic of Internal Medicine, İzmir, Turkey; 4 Katip Çelebi University Faculty of Medicine, Atatürk Training and Research Hospital, Clinic of Radiology, İzmir, Turkey; 5 Katip Çelebi University Faculty of Medicine, Atatürk Training and Research Hospital, Clinic of Pathology, İzmir, Turkey; 6 Bozyaka Training and Research Hospital, Clinic of Radiology, İzmir, Turkey

**Keywords:** B-Cell neoplasms, Acute lymphoblastic leukemias, Acute leukemia

## TO THE EDITOR

**Figure 1:**
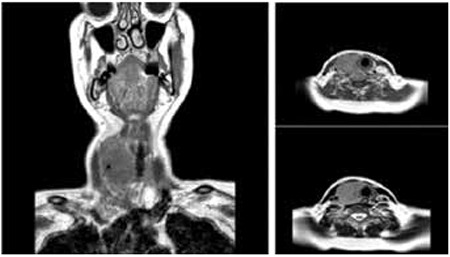
a, b, c: Neck MRI: Enlargement of the right lobe of the thyroid, 5x4.5x3.5 cm sized T1W hypointense, T2W hyperintense nodular lesion occupying right lobe with homogeneous contrast uptake.

Extramedullary presentations of acute lymphoblastic leukemia (ALL) in the central nervous system (CNS), lymph nodes, gonads, spleen, and liver can also be observed. Thyroid infiltration of ALL is very rare. A 53-year-old woman was admitted to the endocrinology outpatient clinic with a lump in the throat, which increased in size over a week. Her medical and family history were nonspecific. Except sensitive and painful thyroid, physical examination findings were normal. Complete blood count values were as follows: Hg 131 g/L, WBC 10,200x109/L, ANC 5490x109/L and platelets 188x109/L. Sedimentation and C-reactive protein (CRP) were higher than normal range. Thyroid hormone levels were within normal range. Antithyroid peroxidase was negative. Thyroid ultrasonography showed moderate enlargement of bilateral thyroid lobes, the parenchyma was hypoechoic and nonhomogeneous. Fine needle aspirates from the thyroid revealed a few lymphocyte and few polymorphonuclear leukocyte infiltration on a necrotic floor with acute inflammation. The neck MRI showed enlargement of the right lobe and a 5x4, 5x3.5 cm, T1W hypointense, T2W hyperintense nodular lesion in the right lobe that was contrasted homogeneously (Figure 1). She was diagnosed with subacute thyroiditis and treated with 1 mg/kg/day methylprednisolone. Three weeks later, she was admitted to hospital with fever, weakness, and enlarging painful thyroid lobes. Physical examination showed pallor, enlarged and painful thyroid lobes, and mild splenomegaly. Complete blood count revealed Hb of 88 g/L, WBC of 8170x109/L, ANC of 2100x109/L and a platelet count of 22x109/L, LDH: 3119. Peripheral blood smear test revealed increased blasts (52% of cells). Bone marrow biopsy showed 80% cellularity with a diffuse, uniform infiltration of lymphoblastic cells with prominent nucleoli. Immunohistochemical staining was positive for TdT, HLA-DR, CD19, CD20, CD22, CD10 and CD38. Chromosome analysis showed 46XX. The breakpoint cluster region-abelson gene (BCR-ABL) fusion was found to be negative. The patient received induction chemotherapy with Berlin-Frankfurt-Munich (BFM) protocol following the diagnosis of precursor-B cell-ALL. In a week pain and enlargement of the thyroid partially regressed, but pain on palpation persisted. On day eight, the second FNA was performed. Pathological results were consistent with leukemic infiltration in a background of very scant colloid. After remission induction therapy, bone marrow aspiration and biopsy showed a continuous rise in diffuse blast cells. During the salvage therapy the patient died due to progresive disease. Subacute thyroiditis is a spontaneously remitting inflammatory condition of the thyroid gland. The thyroid gland is typically enlarged two or three times the normal size and is tender to palpation. When we evaluated the patient retrospectively, we thought that the complaints due to increased thyroid volume were related to leukemic infiltration of the thyroid. Her initially normal complete blood count evolved into pancytopenia. In the literature, there are ALL patients presenting with extramedullary infiltration signs and complete blood count within normal ranges [1,2]. To the best of our knowledge this is the fourth case of B ALL with extramedullary thyroid infiltration in the literature [3,4,5]. As highlighted by the present report, performing fine-needle aspiration cytology should always be considered in the clinical context of a rapidly growing thyroid mass under treatment, and without resolving symptoms.

**Conflict of Interest Statement**

The author of this paper has no conflicts of interest, including specific financial interests, relationships, and/or affiliations relevant to the subject matter or materials included.
